# Supplementary material for: Gut Fungal Communities Are Influenced by Seasonality in Captive Baikal Teal (Sibirionetta formosa) and Common Teal (Anas crecca)
Source: Animals (Basel). 2023 Sep 17;13(18):2948. doi: 10.3390/ani13182948 (PMC10525870; doi:10.3390/ani13182948)
Supplement: Supplementary file 1 [file animals-13-02948-s001.zip › animals-2573308-supplementary.pdf]

animals

Article

Gut Fungal Communities Are Influenced by Seasonality in Captive Baikal Teal (*Sibirionetta formosa*) and Common Teal (*Anas crecca*)

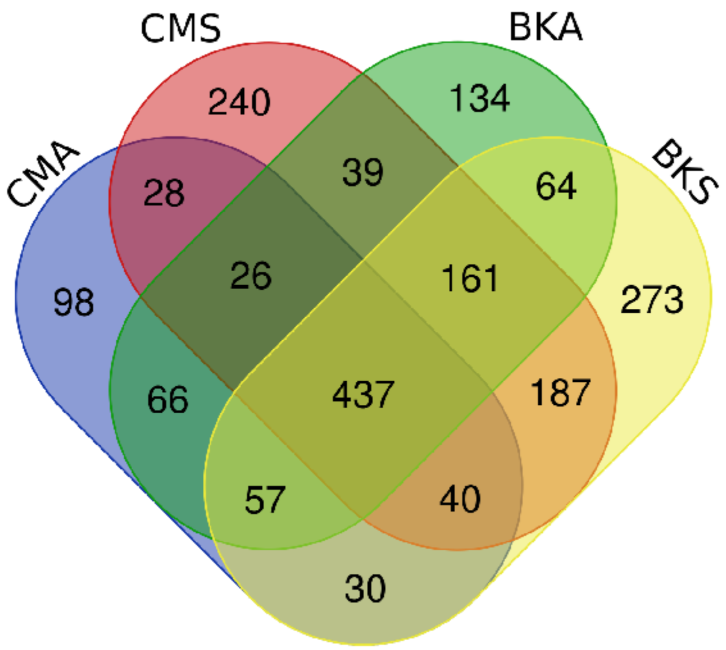

Figure S1. Venn diagram based on OTU level of fungal community within four groups.

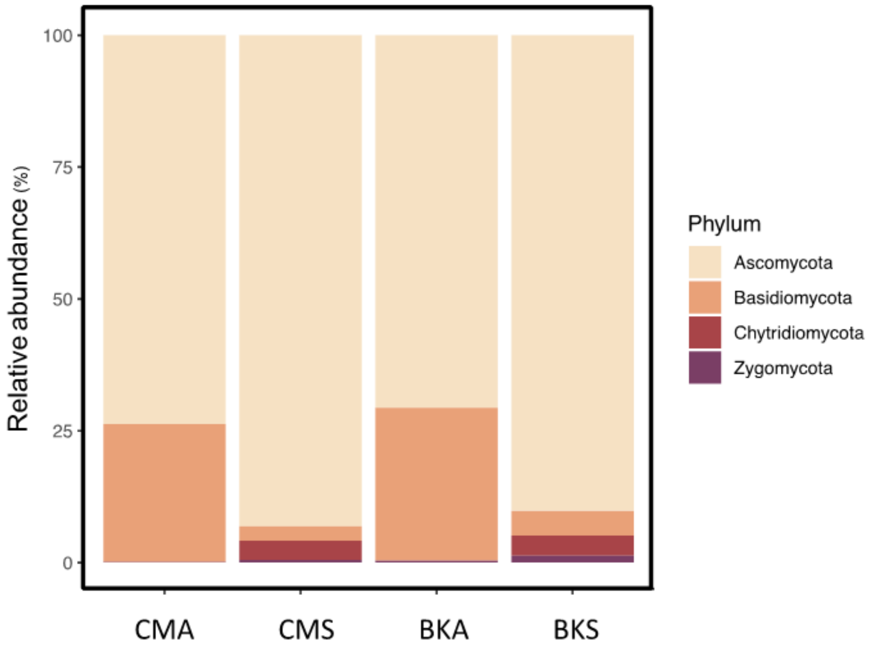

Figure S2. The relative abundance of fungal community in captive Common teal and Baikal teal within summer and autumn at the phylum level.

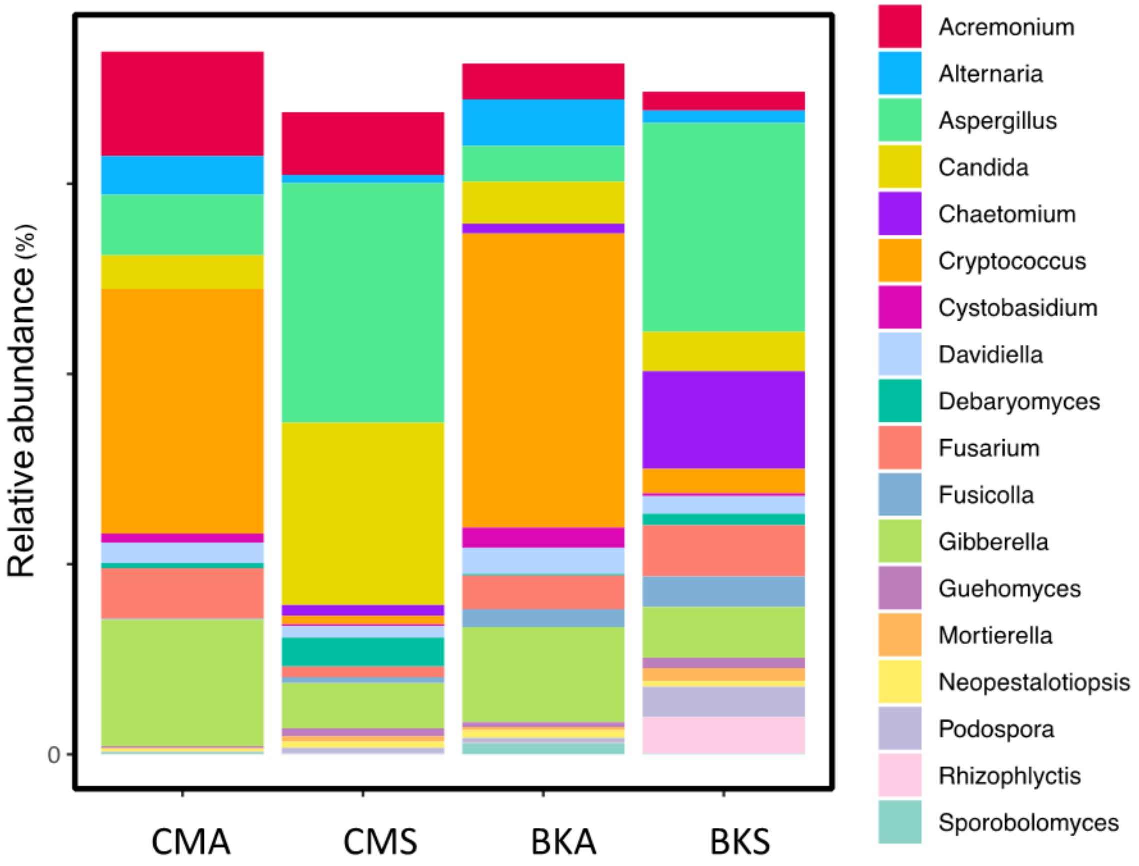

Figure S3. The relative abundance of fungal community in captive Common teal and Baikal teal within summer and autumn at the genus level.

Table S1. The relative of fungal community in captive Common Teal and Baikal Teal within summer and autumn at the phylum level.

Phylum	CMA	CMS	BKA	BKS	Total
Ascomycota	73.73	93.08	70.67	90.14	81.90
Basidiomycota	26.17	2.78	28.93	4.72	15.65
Chytridiomycota	0.02	3.60	0.11	3.82	1.89
Zygomycota	0.09	0.53	0.30	1.31	0.56

Table S2. The relative abundance of fungal community in captive Common teal and Baikal teal within summer and autumn at the genus level.

Genus	CMA	CMS	BKA	BKS	Total
Acremonium	4.70	13.74	2.45	8.25	8.22
Alternaria	6.13	5.07	1.64	1.07	3.92
Aspergillus	4.73	8.00	27.49	31.54	20.23
Candida	5.50	4.41	5.17	23.95	11.00
Chaetomium	1.31	0.02	12.84	1.44	4.40
Cryptococcus	38.65	32.13	3.17	1.05	21.14
Cystobasidium	2.67	1.20	0.42	0.29	1.29
Davidiella	3.47	2.70	2.31	1.50	2.81
Debaryomyces	0.18	0.66	1.51	3.78	1.73
Fusarium	4.43	6.66	6.73	1.39	5.42
Fusicolla	2.33	0.13	4.00	0.83	2.05
Gibberella	12.54	16.61	6.69	5.92	11.77
Podospora	0.65	0.07	4.03	0.77	1.56
Rhizophlyctis	0.04	0.00	4.78	0.02	1.37

Table S3. The ANOSIM test revealed statistically significant differences in the communities of animal pathogens and saprotrophs between the groups.

group	Animal pathogens		Saprotrophs	
	R	Significant	R	Significant
BKA vs BKS	0.1353	0.003	0.1976	0.001
CMA vs CMS	0.4973	0.001	0.3696	0.001
BKA vs CMA	0.0684	0.075	0.8417	0.041
BKS vs CMS	0.1159	0.022	0.0130	0.296

Table S4. The dominant genera of animal pathogens were identified with a considerable relative abundance > 0.01%.

Taxon	BKA	BKS	CMA	CMS
Candida	3.49	3.72	3.89	16.31
Didymella	0.36	0.03	0.48	0.31
Malassezia	0.00	0.02	0.00	0.00
Beauveria	0.00	0.00	0.02	0.01
Engyodontium	0.00	0.00	0.01	0.02

Table S5. The dominant genera of saprotrophic fungi were identified with a considerable relative abundance > 0.1%.

Genus	BKA	BKS	CMA	CMS
Acrocalymma	0.00	0.31	0.00	0.00
Aphanoascus	0.00	0.00	0.00	0.48
Capnobotryella	0.34	0.23	0.00	0.25
Catenaria	0.00	0.24	0.00	3.53
Davidiella	2.23	1.48	2.08	2.77
Debaryomyces	0.11	1.08	0.20	1.09
Guehomyces	0.39	0.97	0.19	0.25
Lasiosphaeriaceae	0.00	0.10	0.00	0.00
Lophiostoma	0.00	0.20	0.00	0.00
Myrothecium	0.12	0.00	0.13	0.18
Neurospora	0.10	0.00	0.00	0.00
Ophiosphaerella	0.00	0.59	0.00	0.00
Paraconiothyrium	0.00	0.42	0.00	0.00
Paraphaeosphaeria	0.00	0.42	0.00	0.00
Penicillium	0.42	0.56	0.20	0.64
Rhodosporidium	0.32	0.20	0.00	0.00
Rhizophlyctis	0.00	3.50	0.00	0.00
Sporormiaceae	0.00	0.00	0.25	0.00
Talaromyces	0.26	0.11	0.08	0.64
Wickerhamomyces	0.00	0.37	0.00	0.39
Zasmidium	0.00	0.00	0.00	0.10
